# Cost-effectiveness of introducing a rotavirus vaccine in developing countries: The case of Mexico

**DOI:** 10.1186/1471-2334-8-103

**Published:** 2008-07-29

**Authors:** Atanacio Valencia-Mendoza, Stefano M Bertozzi, Juan-Pablo Gutierrez, Robbin Itzler

**Affiliations:** 1 Division of Health Economics, National Institute of Public Health, Av. Universidad No. 655, Col. Santa María Ahuacatitlán, Cuernavaca, Morelos, 62508, México; 2 Center for Evaluation Research and Surveys, National Institute of Public Health, Av. Universidad No. 655, Col. Santa María Ahuacatitlán, Cuernavaca, Morelos, 62508, México; 3 Haas School of Business, University of California, Berkeley, USA; 4 Center for Economic Education and Research (CIDE), Mexico City, México; 5 Division of Health Surveys, National Institute of Public Health, Av. Universidad No. 655, Col. Santa María Ahuacatitlán, Cuernavaca, Morelos, 62508, México; 6 Merck & Co., Inc., UG1C-60, P.O. Box 1000, North Wales PA 19454-1099, USA

## Abstract

**Background:**

In developing countries rotavirus is the leading cause of severe diarrhoea and diarrhoeal deaths in children under 5. Vaccination could greatly alleviate that burden, but in Mexico as in most low- and middle-income countries the decision to add rotavirus vaccine to the national immunisation program will depend heavily on its cost-effectiveness and affordability. The objective of this study was to assess the cost-effectiveness of including the pentavalent rotavirus vaccine in Mexico's national immunisation program.

**Methods:**

A cost-effectiveness model was developed from the perspective of the health system, modelling the vaccination of a hypothetical birth cohort of 2 million children monitored from birth through 60 months of age. It compares the cost and disease burden of rotavirus in an unvaccinated cohort of children with one vaccinated as recommended at 2, 4, and 6 months.

**Results:**

Including the pentavalent vaccine in the national immunisation program could prevent 71,464 medical visits (59%), 5,040 hospital admissions (66%), and 612 deaths from rotavirus gastroenteritis (70%). At US$10 per dose and a cost of administration of US$13.70 per 3-dose regimen, vaccination would cost US$122,058 per death prevented, US$4,383 per discounted life-year saved, at a total net cost of US$74.7 million dollars to the health care system. Key variables influencing the results were, in order of importance, case fatality, vaccine price, vaccine efficacy, serotype prevalence, and annual loss of efficacy. The results are also very sensitive to the discount rate assumed when calculated per life-year saved.

**Conclusion:**

At prices below US $15 per dose, the cost per life-year saved is estimated to be lower than one GNP per capita and hence highly cost effective by the WHO Commission on Macroeconomics and Health criteria. The cost-effectiveness estimates are highly dependent upon the mortality in the absence of the vaccine, which suggests that the vaccine is likely to be significantly more cost-effective among poorer populations and among those with less access to prompt medical care – such that poverty reduction programs would be expected to reduce the future cost-effectiveness of the vaccine.

## Background

Worldwide, infection with rotavirus is the leading cause of severe diarrhoea in children under age 5. Before age 5, most children will have experienced an episode of rotavirus gastroenteritis. Rotavirus infection may be either asymptomatic or symptomatic. In the latter case, it is associated with acute gastroenteritis, beginning with an acute episode of watery diarrhoea, fever, and vomiting [1,2]. The diarrhoea lasts for an average of six days, although cases have been reported in which it has lasted up to 20 days [3]. Rotavirus infection is typically more severe than other common causes of childhood diarrhoea, and is more likely to be associated with dehydration, hospitalisation, and death [1,4-6]. Some estimates have shown that 20%–40% of all hospital admissions and 20% of deaths from diarrhoea are attributable to rotavirus in children under age 5 [4]. Every year, rotavirus causes approximately somewhere between 352,000 and 592,000 deaths, 2 million hospital admissions, 25 million medical visits, and 111 million cases of gastroenteritis requiring only home care, in children under 5 years old [4]. As a result, 1 out of 5 children with rotavirus gastroenteritis will visit a physician; 1 out of 65 will be admitted to a hospital; and approximately 1 out of 293 will die [4].

In temperate climates, rotavirus diarrhoea appears mostly in winter, whereas in tropical climates seasonal patterns of the disease are less evident [7] Incidence of rotavirus disease is similar among children in developed and developing countries. However, children in developing countries have higher mortality rates due to several factors, including less access to oral rehydration and a higher prevalence of malnutrition. An estimated 1,205 children die from rotavirus disease every day, and 82% of those deaths occur among children in low-income countries. In fact, children in low-income countries are nearly 3, 6 and 237 times as likely to die from rotavirus as children in low middle, upper middle and high income countries, respectively [4] Therefore, the potential benefits of a rotavirus vaccine in low-income countries and in the poorest parts of middle-income countries such as Mexico could be very considerable.

A new pentavalent rotavirus vaccine (PRV) for the prevention of rotavirus gastroenteritis has been developed (RotaTeq^©^, Merck). The vaccine contains five live bovine-human reassortant serotypes (G1, G2, G3, G4, and P1A[8]). The vaccine is given by mouth in a three-dose schedule, starting at 6 weeks of age, with one- to two-month intervals between doses. The vaccine has been proven safe; and when the three-dose schedule is given, its efficacy against G1–G4 rotavirus gastroenteritis of any degree of severity is 74.0% (66.8 to 79.9% 95% CI), whereas efficacy against severe G1–G4 gastroenteritis is 98.0% (88.3 to 100% 95% CI). Taking this into consideration, this vaccine could prevent many of the deaths and hospitalisations resulting from severe episodes of rotavirus gastroenteritis.

Mexico, like many other low- and middle-income countries is currently facing decisions about whether and how to incorporate newly available, expensive vaccines into its national vaccination program. Just within the last two years the government initially decided to make the monovalent vaccine available for a subset of counties with high infant mortality rates and more recently decided to expand vaccination to the entire country.

In most low- and middle-income countries the decision to add a rotavirus vaccine to the national immunisation program will depend heavily on the cost-effectiveness and affordability of the vaccine. We present here a model developed for such settings and projections from the model using Mexican data.

## Methods

### Target population and perspective

A cost-effectiveness model was developed to inform the decision about whether to include universal vaccination with PRV in a national immunization program.

The cost-effectiveness analysis compares the cost of treatment for rotavirus diarrhoea in the absence of a national immunisation program with the cost of a national immunisation program plus the cost of treatment for those cases not averted by immunisation. Three doses of PRV would be given by mouth during the first 6 months of life. Three cost-effectiveness ratios are estimated: the incremental cost of the program divided by (1) the number of cases prevented, (2) the number of deaths prevented, and (3) the life-years gained. Cost-effectiveness is estimated from the perspective of the health care system, taking into account both the medical costs associated with rotavirus diarrhoea (as if all medical care costs were borne by the system) and the costs of the immunisation program. Productivity benefits and costs or savings that accrue to families (e.g. transport and childcare) other than medical care were not included.

In the application of the model to Mexico, an annual birth cohort of two million children is assumed. Given the seasonality of rotavirus disease, the birth cohort is divided into 12 monthly sub-cohorts to distribute the births throughout the year, assuming a constant number of births per month. Costs are presented in 2006 dollars. All future costs and health benefits are discounted at 3% in the base case. Rotavirus-associated medical costs are estimated for the first 5 years of life using age-specific incidence by season for each event.

### Decision analysis model

**Figure 1:**
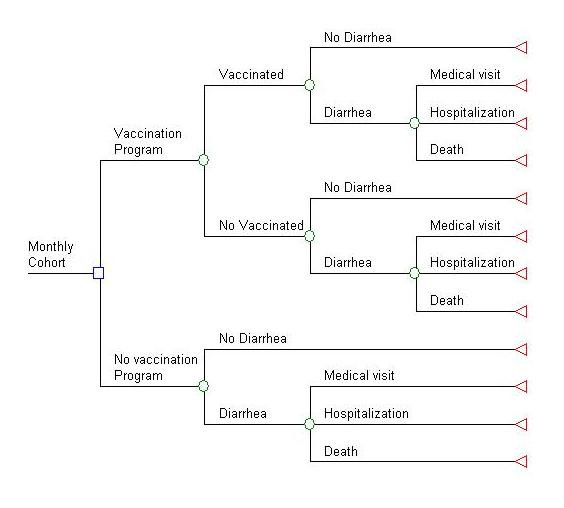
Schematic representation of the model for rotavirus immunisation program in Mexico.

The costs and benefits of implementing a national rotavirus immunisation program were compared with a probabilistic model using spreadsheet based software (@RISK 4.5.4; Pallisade, Newfield, NY) in EXCEL (Figure 1). The model was analyzed under baseline conditions in order to determine the costs of either option: immunizing at the coverage levels of diphtheria and tetanus toxoids and pertussis (DTP) vaccine, or the prevailing situation, with no vaccine. Key variables used in the model were varied using univariate and probabilistic sensitivity analysis (see Tables 1 through 4).

**Table 1:** Disease parameters

	**Monthly morbidity from rotavirus diarrhoea as a percentage of diarrhoea by all causes, 2002^1^**	**Monthly morbidity from diarrhoea (per 100,000), 2003^2^**			**Monthly mortality from diarrhoea (per 100,000), 2002^3^**		
			
	Under age 1	Under age 1	Age 1 to 4	Birth to age 5	Under age 1	Age 1 to 4	Birth to age 5
Jan	62.6	289.3	228.6	296.4	10.88	1.2	3.1
Feb	66.95	228.6	226.4	228.6	7.11	0.7	2
Mar	70.43	217.9	285.7	226.4	6.22	0.7	1.8
Apr	63.47	296.4	325	285.7	4.66	0.5	1.3
May	46.08	342.9	321.4	325	5.66	0.6	1.6
Jun	26.08	328.6	457.1	321.4	4	0.4	1.1
Jul	1.73	482.1	335.7	457.1	5.33	0.6	1.5
Aug	0.43	357.1	375	335.7	5.44	0.6	1.6
Sep	8.69	300	296.4	375	4.22	0.4	1.1
Oct	6.08	314.3	200	296.4	3.55	0.4	1
Nov	29.56	225	150	200	4.77	0.5	1.3
Dec	63.47	164.2	100	100	8	0.8	2.2

1/Source: InDRE (Institute for Epidemiologic Diagnosis and Reference)

2/Source: Health Ministry and SUIVE (Unified Information System for Epidemiological Surveillance)

3/Source: INEGI (National Institute National Institute of Statistics, Geography and Informatics)

**Table 2:** Health services utilization for children under 5 with diarrhoea

	**Hospital admissions**	**Outpatient visits**	Total	% requiring hospitalization
< 1 year	6,390	37,449	43,839	14.58%
1 year	4,250	110,805	115,055	3.69%
2 years	4,997	89,481	94,478	5.29%
3 years	2,367	56,810	59,177	4.00%
4 years	1,459	40,180	41,639	3.50%

Source: Instituto Mexicano del Seguro Social (IMSS), 2005

**Table 3:** Parameters used in the model

	Baseline scenario	Range for sensitivity analysis	Source
*Efficacy for the first year following vaccination (%)*			
Medical visits	87.7	(76.2–93.7)	Vesikari T, et al, 2007
Hospital admissions	96.3	(91.1–98.5)	Vesikari T, et al, 2007
*Efficacy for the second year following vaccination (%)*			
Medical visits	73.8	(11.5–95.7)	Vesikari T, et al, 2007
Hospital admissions	86.9	(10.8–99.7)	Vesikari T, et al, 2007
			
Prevalence of G1–G4 serotypes (%)	90.0	(83–97)	Mota-Hernandez, et al, 2003
			
Cost per medical visit) 2006 dollars)	35.85	(34.59–37.29)	Nafate, 2005
Cost per hospital day (2006 dollars)	222.55	(215.13–229.97)	Nafate, 2005
Cost of the vaccine per dose (2006 dollars)	10.00		
Cost of administration for the whole schedule (2006 dollars)	13.77		
Discount rate	3%		
			
*Life expectancy in years at age:*			
0 years	70.38		‡
1 years	71.95		‡
2 years	71.74		‡
3 years	70.84		‡
4 years	69.91		‡

‡ CONAPO's own estimates. Mortality tables, 2002. Available at

**Table 4:** Variables, values and distributions for the probabilistic sensitivity analysis

Variable	Values	Distribution
Reduction in outpatient visits	Median 0.877 5^th ^percentile 0.762 95^th ^percentile 0.937	Beta
Reduction in hospitalization	Median 0.963 5^th ^percentile 0.911 95^th ^percentile 0.985	Beta
Efficacy between the first and the third dose	Median 0.726 5^th ^percentile 0.574 95^th ^percentile 0.828	Beta
Annual efficacy decrease	Median 0.1 5^th ^percentile 0.05 95^th ^percentile 0.18	Beta
Outpatient visit cost	Median 34.59 5^th ^percentile 34.59 95^th ^percentile 37.29	Gama
Hospitalization day cost	Mean $252.52	Exponential

In this model the current practice with no vaccination is compared with a vaccination program in which pentavalent rotavirus vaccine is included in a national childhood immunisation program at current levels of coverage for DTP. The vaccination program option represents inclusion of PRV in a national immunisation program, for a birth cohort of two million children (followed from birth to 5 years of age) divided into 12 monthly sub-cohorts of the same length. With a vaccination program, some children may not be vaccinated, and because vaccine efficacy is less than 100% against rotavirus diarrhoea, some vaccinated children also will become ill (Figure 1).

Given the perspective adopted in this study, the model does not distinguish between children who do not become ill with rotavirus diarrhoea and those who become ill and do not use healthcare services (unless they die). In other words, the model does not consider the potential benefit associated with preventing cases of diarrhoea that are treated at home. In the diarrhoea arm of the model are those who experience a medical visit, hospitalisation, or death.

In this model the cohort is followed from birth to 5 years of age and is allowed to have different incidence of rotavirus medical visits, hospitalisations and deaths, according to the month of the year and the age, in months, of the children. The medical visits, hospitalisations and deaths averted each month by the program correspond to the reduction of incidence of each outcome due to serotype-specific vaccine efficacy. The advantage of this approach is that it enables the modelled benefits to reflect the lack of vaccine benefit in reducing incidence prior to the first dose, as well as different levels of protection after the first, second and third doses. It also enables estimation of how benefits can vary because of the seasonality of the incidence of the virus. Once data become available in Mexico on the real ages at which children receive the vaccine (as compared to the ideal 2, 4, and 6 month vaccination schedule modelled here) it will be possible to model the expected reduction in benefit associated with delays in vaccination.

The model inputs including disease burden, medical costs, rotavirus serotypes prevalence, discount rate, efficacy, coverage and price of the vaccine are shown in Tables 1 to 4.

### Rotavirus disease: morbidity and mortality

Because the model estimates morbidity and mortality for each monthly sub-cohort, it is possible to estimate the expected number of cases, nationally, for each event in each sub-cohort, by month and age, in order to consider seasonal disease pattern as well as the seasonality of vaccination campaigns when estimating the benefits of the vaccine. In this way the model accounts for potential differences in health benefit when a child is vaccinated just before the beginning of the high-risk season or just after it finishes. This is relatively unimportant for countries that predominantly vaccinate children at routine well-baby visits, but potentially quite significant for countries that vaccinate with annual or semi-annual vaccination campaigns.

The model assumes that serotype specific effectiveness of PRV is constant within a country or region of interest. This implies that the proportionate reduction of hospitalisations and outpatient visits for any particular serotype is assumed to be the same everywhere. This is supported by a recent analysis of the Rotavirus Efficacy and Safety Trial (REST) by region [8]. However, there are large differences among countries in the proportion of illness due to different serotypes as well as differences in severity of illness given underlying differences in nutritional and health status as well an availability of prompt and effective care for children with diarrhoea. Thus, use of the model in different settings requires calibrating it to local serotype incidence, and age-specific disease incidence and severity. In Table 3 we present the prevalence of G1–G4 serotypes specific for Mexico used in the model.

In the case of Mexico, nationally representative data are available for diarrhoea incidence for 0 – 11 months of age and for 12 – 59 months of age as well as the proportion of diarrhoea in the 0 – 11 month group that is due to rotavirus. However, there is no further breakdown by age of the diarrhoea incidence for Mexico. The IMSS, the Mexican Social Security system that cares for more than 30 percent of the population, has data on incidence by month in the first year of life and by year till age 5. Because the IMSS population is wealthier on average than the non-IMSS population, the overall incidence of severe disease is lower, but the relative distribution of disease among the months/years is probably similar to that in the general population. Thus, the IMSS data were used to distribute incident cases among the months of year 1 and amongst the years 1–4 as required for the model (see Table 2).

There is also no information about how the proportion of diarrhoea cases due to rotavirus changes with age in Mexico. For children less than 1 year of age we only had the overall proportion of diarrhoea due to rotavirus so we used the distribution from the Marie-Cardine *et al *study from France to impute the distribution across the 12 months [9]. This distribution is critical because it enables quantification of changes in benefit associated with delays in vaccination due, for example, to campaign vaccination as opposed to routine vaccination. Mexican data are not available on the proportion of diarrhoea cases due to rotavirus among children one year or older, thus we used the proportions reported by Rivest *et al *from Canada, normalised to the overall proportion due to rotavirus in Mexico [10]. The normalisation was done comparing the overall proportion due to rotavirus 0–12 mo in Canada with that in Mexico.

Table 1 shows the monthly morbidity from rotavirus diarrhoea in children under age 1 as a percentage of all cases of diarrhoea during 2002. The source for these estimates was a study carried out in secondary- and tertiary-care sentry hospitals in 15 large cities across Mexico, with data from 1996 through 2002 [11].

Data on the incidence and mortality associated with diarrhoea from all causes, as well as the fraction requiring medical visits or hospital admission, obtained either monthly or by age groups from various sources, are shown in Tables 1 and 2.

In each monthly sub-cohort, representing infants born during 2006, the expected numbers of medical visits, hospital admissions and deaths attributable to rotavirus were estimated for every month of life up to the age of 5 (60 months). These estimates were based on the specific incidence by month and age group of events attributable to rotavirus (see Tables 1 and 2). Even though rotavirus diarrhoea is reported to be more severe on average than the other major causes of childhood diarrhoea, no Mexican data are available on how the proportion attributable to rotavirus differs among cases treated at home, cases treated in ambulatory settings, cases hospitalized, and deaths. The situation is further complicated by the fact that initial infection with rotavirus is likely to be more severe than subsequent infections, even by other serotypes [12]. To the extent that this is true, then the relative severity of rotavirus is likely to fall with age. In absence of data, we assumed that the proportion of diarrhoea cases due to rotavirus was constant across health care settings.

### Vaccine coverage levels, efficacy estimates, and prevalence of G1, G2, G3, and G4 serotypes in Mexico

The vaccine coverage for PRV is anticipated to be the same as the one for the DTP vaccine, since both have a recommended schedule of administration at 2, 4, and 6 months of age. In 2004, the coverage of the full schedule of DTP vaccine was 94% in children under age 1 [13]. In the baseline scenario, we assume that the vaccine will be given during the first 6 months of life as recommended, with the three doses at 2, 4, and 6 months of age. Alternatively, to see how sensitive the results are to delays in vaccination, we estimated a scenario in which children are vaccinated at 6, 8 and 10 months of age (maximum age permitted).

The efficacy estimates were taken from a clinical trial of PRV [8], containing five live human-bovine reassortant rotavirus strains (G1, G2, G3, G4, and P1A[8]). Efficacy estimates in this study show an 87.7% reduction in office visits from gastroenteritis caused by serotypes G1, G2, G3, and G4 (95% CI, 76.2–93.7), and a 96.3% reduction in hospital admissions from gastroenteritis caused by serotypes G1, G2, G3, and G4 (95% CI, 91.1–98.5, Table 3). Efficacy against deaths from rotavirus gastroenteritis is assumed to be similar to efficacy in reducing hospitalisations. In the sensitivity analysis, the efficacy of the vaccine was varied within the range of its confidence interval in the clinical trial. These assumptions are conservative. The emergency room visits were combined with outpatient visits assuming the latter's efficacy, despite trial data suggesting a greater reduction in the more severe disease treated in emergency rooms because no information was available on the number of emergency room visits in Mexico. Similarly, one would expect deaths to be reduced more than hospitalisations.

The aforementioned efficacy estimates correspond to the first year starting 14 days after completion of the three dose regimen. Efficacy estimates are also reported for the second year, showing a slight decrease in protection against the disease (Table 3). To take into account the likelihood of decreasing protection conferred by PRV against rotavirus disease over time, and the lack of data on protection after the second year, the baseline scenario assumes an annual 10% efficacy reduction from year three to five. In the univariate sensitivity analysis, this percentage was varied from 0 to 30%.

The vaccine clinical trials for PRV were powered to estimate the protection conferred by the full vaccination schedule and not the partial protection conferred after the first or second dose. However, the model must consider the effect of the vaccine before all three doses are administered. Two different efficacy estimates are available from the clinical trial: that from the intent-to-treat analysis, which includes all enrolled children, whether they received the full vaccine schedule or not, and the per-protocol analysis which only considers those who received the three doses per protocol. We considered the intent-to-treat efficacy as the weighted average of the efficacy for full schedule and the efficacy for partial schedule and used that to estimate the effectiveness of a partial schedule. The estimation procedure was as follows:

In algebraic terms we have:

E_PP _*(P_PP_/P_ITT_)+X*(1-P_PP_/P_ITT_) = E_ITT_

Where:

E_PP _= efficacy for the first year in the per-protocol study

P_PP _= subjects in the per protocol study

P_ITT _= subjects in the intent-to-treat study

E_ITT _= efficacy for the first year in the intent-to-treat study

X = efficacy between the first and the third dose, which is the unknown variable we want to estimate

Note that P_PP_/P_ITT _is the weight corresponding to the subjects who received all three doses among all subjects enrolled, and that (1-P_PP_/P_ITT_) is the weight of those with an incomplete immunisation schedule. This approach doesn't provide information about how protection is different between the first and second dose from that between the second and the third. We have assumed constant effectiveness between the first and third dose.

Finally, the expression corresponding to the estimation is

X = (E_ITT_-E_PP _*(P_PP_/P_ITT_))/(1-P_PP_/P_ITT_)

The reported efficacy estimates relate to protection against disease caused by serotypes G1, G2, G3, and G4. To estimate the potential reduction in the number of target events (medical visits, hospitalisations, and deaths) achieved by the vaccine, the fraction of rotavirus gastroenteritis attributable to serotypes G1, G2, G3, and G4 will be taken into account; in Mexico, this figure is 90% [14]. In the sensitivity analysis, this percentage was varied from 78 to 92%, to account for the range reported in the literature.

### Cost estimates

Medical costs include the costs of outpatient visits and hospital admissions, as well as the costs associated with immunisation. To reflect the heterogeneity of the Mexican Health System we considered the cost of illness estimated for the main health institutions. We used the costs estimated by Náfate-Martínez (2005) for the Ministry of Health, the IMSS (Mexican Social Security Institute), and private providers [15]. These system-specific estimates were averaged by weighting each by the proportion of people who receive treatment nationally from that system. The estimate for the IMSS was assumed to be representative of all social security institutions (it is by far the largest). Table 3 shows data on the estimated costs per outpatient visit and hospital day from gastroenteritis converted into US dollars at 10.89 pesos per dollar the exchange rate for September 8th, 2006. The average length of a hospital stay was estimated to be five days. In the probabilistic sensitivity analysis, cost per medical visit was varied using a gamma distribution and the cost per hospitalization using an exponential distribution (Table 4). For the univariate sensitivity analyses, the upper and lower bounds of the 95% confidence interval were used.

For our baseline scenario, the price for each dose of the vaccine was set at $10 dollars. Furthermore, the cost for administering the full immunisation schedule, excluding the vaccine itself, was assumed to be 150 Mexican pesos and converted into US dollars at 10.89 pesos per dollar the exchange rate for September 8^th^, 2006.

Given the chosen perspective, we have only considered medical costs associated with either of the two alternatives (immunisation and no immunisation). A further analysis could encompass a societal perspective, including non-medical costs for both alternatives, such as transportation costs and productivity losses.

### Life-years saved

Once the number of deaths preventable by immunisation in our cohort was estimated, the life-years saved were also estimated, based on the mortality tables published by CONAPO (the Mexican National Population Council), from which age-specific life expectancies were obtained (Table 3) [16]. Life-years saved by the intervention were calculated as the sum of deaths preventable at each age, multiplied by life expectancy at that age, which were discounted at a three percent rate.

### Sensitivity analysis

#### Univariate sensitivity analysis

Starting from the baseline scenario, univariate sensitivity analyses were carried out to examine the extent to which the uncertainty in the variables affects our estimates. Considering that a market price for the vaccine has not been set, a sensitivity analysis on this variable was also performed. This makes it possible to anticipate cost-effectiveness values for the program in a range of potential scenarios of price per dose.

Given that the rotavirus case-fatality rate changes over time (improvements in health care access, nutrition, poverty alleviation programs, etc.), we performed a separate analysis varying the actual case-fatality rate from 50% to 200% to see how sensitive the cost per life-year saved is throughout these different contexts.

#### Probabilistic sensitivity analysis

To explore the robustness of the model and to estimate a feasible variation range of the cost-effectiveness results we conducted probabilistic sensitivity analyses and ran a hundred thousand model iterations with random sampling from the distributions.

For each of the uncertain variables in the model we defined a range and distribution of variation for the probabilistic sensitivity analysis. Table 4 shows the variables used in the probabilistic sensitivity analysis, their values and assumed distributions.

Because the vaccine price has not yet been set by the manufacturer, the model was also estimated for across a range of possible prices from $5 to $15 dollars per dose.

## Results

### Baseline scenario

Our estimates suggest that if Mexican children were vaccinated as part of a national immunisation program, universal vaccination could prevent 71,464 medical visits (59%), 5,040 hospital admissions (66%), and 612 deaths from rotavirus gastroenteritis (70%) (Table 5). Medical costs for rotavirus diarrhoea estimated for the cohort were 11.9 million dollars without the immunisation program. Assuming a $10 dollar price per dose, the national immunization program would cost $82.3 million dollars, and would save $7.6 million dollars in medical costs. Thus, the national immunization program would have a net cost of $74.7 million dollars, leading to a net cost per death prevented (or life saved) of $122,058 dollars and a cost per discounted life-year saved of $4,283 dollars (Table 5).

**Table 5:** Results in terms of health and costs, with and without the rotavirus vaccine program

**Variable**	**Without the program**	**With the program**	**Preventable by the vaccine** (5^th^–95^th ^percentile)*	**% reduction**
Medical visits	122,134	50,670	71,464 (68,135 – 74,432)	59%
Hospital admissions	7,585	2,545	5,040 (4,851 – 5,186)	66%
Deaths	871	259	612 (593 – 628)	70%
Life-years saved			43,411 (42,092 – 44,570)	
Discounted Life-years saved			17,430 (16,888 – 19,908)	
				
**Medical costs (thousands of dollars)**				
For medical visits	$ 4,046.74	$ 1,671.90	$ 2,374.84 (2,134 – 2,638)	59%
For hospital admissions	$ 7,905.79	$ 2,649.22	$ 5,256.57 (293 – 17,825)	66%
Cost of the vaccine		$ 56,400.00	-$56,400.00 (NA)	
Cost of administration of the vaccine		$ 25,895.32	-$25,895.32 (NA)	
**Total medical costs**	**$11,952.53**	**$ 86,616.44**	**-$74,663.91** ** (-79,624 – -62,049)**	
				
**Cost per case prevented (dollars)**	**$ 968.23** ** (796 – 1,054)**			
**Cost per hospitalization prevented (dollars)**	**$ 14,814.88** ** (12,277 – 16,045)**			
**Cost per death prevented (dollars)**	**$122,058.40** ** (101,113 – 131,226)**			
**Cost per life-year saved (dollars)**	**$ 1,719.93** ** (1,424 – 1,848)**			
**Cost per life-year saved (dollars, Discounted life-years)**	**$ 4,283.75** ** (3,548 – 4,606)**			

* 5^th ^to 95^th ^percentile of the probabilistic estimates

### Univariate sensitivity analysis

**Figure 2:**
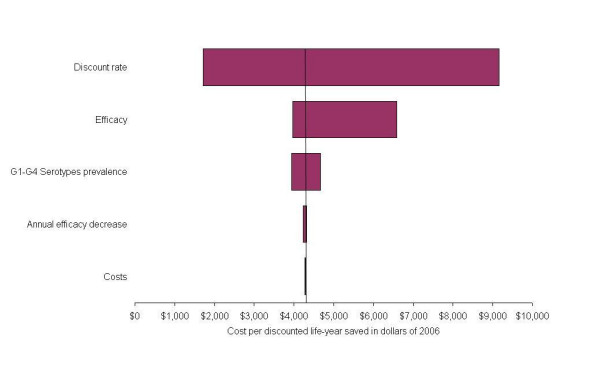
Univariate sensitivity analysis.

The leading determinants of the cost-effectiveness results were, case fatality, vaccine price, vaccine efficacy, serotype prevalence, and annual loss of efficacy. When our model was evaluated at the lower limit of the efficacy parameters, the cost per life-year saved rose from $4,283 to $6,598 dollars (+54%), whereas at the upper limit, our cost-effectiveness ratio falls to $3,969 dollars (-7.3%) per life-year saved (Figure 2). The asymmetry reflects the fact that in the base case the effectiveness is very high and has little room to improve.

On the other hand, our estimates are less sensitive to the medical costs of the disease. Varying the estimated costs by the width of the confidence interval leads to a -0.3% to +0.4% variation in the cost per life-year saved (Figure 2). The results are very sensitive to the discount rate assumed when calculated per life-year saved because all costs are incurred in the first couple of years of life, but benefits continue for a lifetime. The model is not sensitive to discount rate when calculated per case of diarrhoea, hospitalisation or death averted because the costs and benefits occur concurrently.

**Figure 3:**
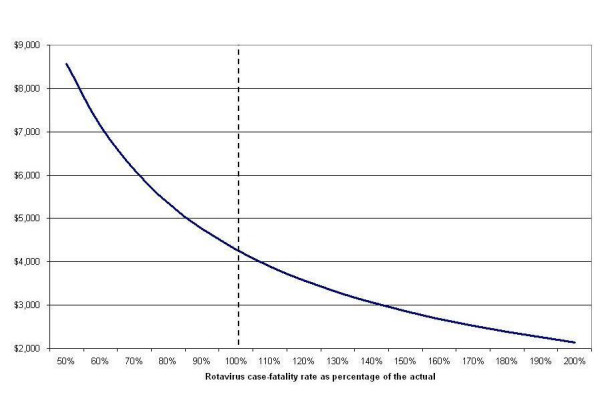
Relationship between rotavirus case-fatality rate and cost per discounted life-year saved (US dollars of 2006).

When we calculated per life-year saved, our cost-effectiveness estimates are highly sensitive to variations in case-fatality rate. Assuming a case-fatality rate 50% lower than the actual rate, the model yields a cost per life-year gained of US$8,566 (a 100% increase), and assuming a case-fatality-rate twice the actual rate, the cost per life-year gained is US$2,142 (a 50% decrease) (Figure 3).

When assuming a scenario in which children are vaccinated at 6, 8 and 10 months of age, deaths averted fell to 501 (a 18% decrease), while the cost per discounted life-year saved increased to US$5,292 (a 19% increase).

### Probabilistic sensitivity analysis

**Figure 4:**
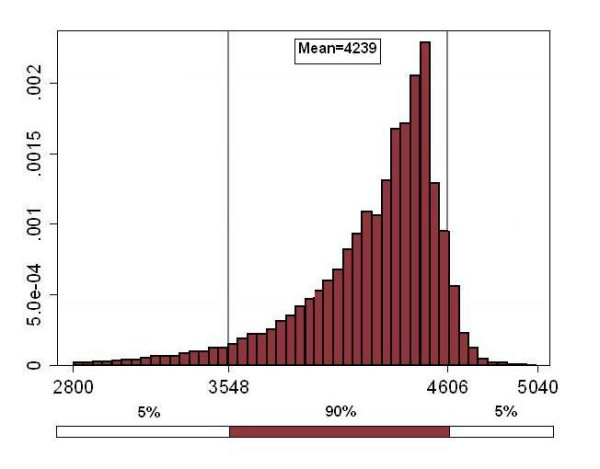
Cost per discounted life-year saved (US dollars of 2006).

For a price scenario of $10 dollars per dose we ran a hundred thousand iterations using the parameter distributions defined in Table 4. The output for cost per discounted life-year saved is graphed in Figure 4. Ninety percent of the resulting cost-effectiveness estimates fell in the interval of $3,548 to $4,606 dollars per discounted life-year saved.

The results for the rest of the output variables are presented in brackets in Table 5 for the 5^th ^and 95^th ^percentile of the estimates. Ninety percent of the estimated averted deaths fell in the interval of 593 to 628. The same interval for discounted life-years saved was 16,888 to 19,908 and for net cost it was 62 to 79.6 million dollars.

**Figure 5:**
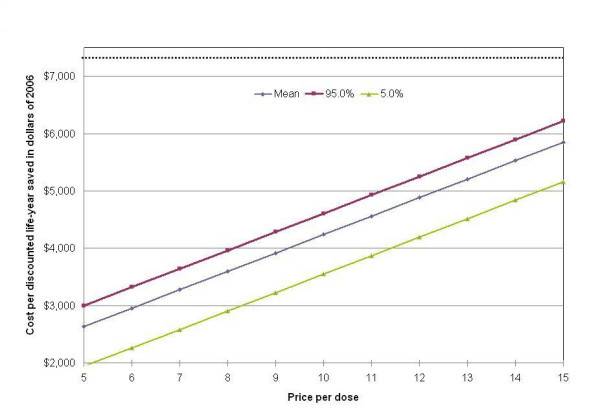
Probabilistic sensitivity analysis.

Figure 5 presents the results of the same sensitivity analysis conducted across the range of prices per dose. The shaded area bounded by the dotted lines is the area in which ninety percent of the estimates fall, for each price. Given that there is no correlation between vaccine price and the uncertainty of any of the other parameters, the size of the interval does not vary with changes in price. The horizontal line at $7,310 represents the 2005 Mexican GNP per capita in US dollars, as point of reference.

## Discussion

At prices below US $15 per dose, using base-case parameter values, the cost per life-year saved is estimated to be lower than one GNP per capita. This is a very conservative estimate because it ignores the benefits associated with preventing non-fatal cases of rotavirus, with the exception of the savings in health care costs. Given the absence of other strategies to prevent rotavirus infection and the fact that Mexico has already widely implemented a national public health program using oral rehydration therapy to reduce deaths from childhood diarrhoea, there was no obvious alternative intervention against which vaccination can be compared. However, the WHO Commission on Macroeconomics and Health suggested that interventions are highly cost-effective if the cost per life-year saved is lower than the per capita GDP; may be cost-effective if their cost is less than two to three times the per capita GDP; and are cost-ineffective if their cost is higher than three times the per capita GDP [17]. This suggests that a rotavirus immunisation program would be highly cost-effective across a wide range of price-per-dose scenarios.

We estimated that delaying vaccination four months could reduce by 18% the number of deaths prevented with respect to vaccinating at the recommended ages. This result gives us some lights of the expected reduction in effectiveness for countries where most vaccination is conducted in campaigns where delays are caused by waiting till the next campaign.

The most common manner in which morbidity and mortality are simultaneously considered is via cost-utility analysis, using a measure of effectiveness such as quality adjusted life years (QALYs). However, in the case of rotavirus infection it would not be difficult to argue that the impact of the illness on parental leisure and productivity is a greater burden for households than the decrement in the quality of life of the infant. As a result, a willingness to pay study might be more useful for capturing the value of benefits of vaccination beyond mortality than a cost-utility analysis.

As with any vaccine, estimates of cost-effectiveness are highly dependent upon the expected morbidity and mortality in the absence of the vaccine. The estimates presented here reflect Mexican national averages in rotavirus-associated morbidity and mortality. While the incidence of infection with rotavirus is not strongly correlated with socioeconomic status, the likelihood that an infection results in severe dehydration requiring hospitalisation, or in death, is strongly negatively correlated with socioeconomic status. This suggests that the vaccine is likely to be significantly more cost effective among poorer populations and among those with less access to prompt medical care. It also suggests that poverty reduction programs, nutritional support programs and the introduction of health insurance programs for the poor, such as the ones recently introduced in Mexico would be expected to reduce the cost-effectiveness of the vaccine in the future.

As with any modelling exercise, the necessary simplification of a complex reality implies limitations that must be considered in the application of the results of the modeling exercise to public policy. In almost every case the simplifications reflected in this model would tend to underestimate the true cost-effectiveness of the vaccine. The exception is that the model does not consider possible vaccine side effects that might become apparent with large-scale implementation of the vaccine even though they were not observed in the clinical trial. Although none of the clinical trials or post-market surveillance to date suggest that this vaccine has significant vaccine-related mortality or morbidity, there always exists the possibility that a very low frequency side effect has not yet been detected – as occurred with RotaShield. However, given the high burden of Rotavirus disease in Mexico and other developing countries, even if this were to occur it is very unlikely to significantly alter the cost-effectiveness of the new vaccine.

As mentioned above, the model does not consider the impact of illness on parental productivity or the value to households of averted illness. The cost of illness is likely to be underestimated because it included outpatient and inpatient costs, but did not separately estimate costs of emergency care. In addition, the sample of facilities used for costing did not include any tertiary-care facility.

The model assumes that children who do not die of rotavirus infection have average age-specific life expectancy. To the extent that children who die of rotavirus are those who are poorer and/or have less access to health services and/or have other health problems including malnutrition, then their life expectance may be shorter and the estimated benefits optimistic. In an upper middle income country such as Mexico, this bias is unlikely to be important, especially because at almost any discount rate the marginal difference in life expectancy is irrelevant. However in low income countries with high levels of competing infant mortality this is likely to be more important.

It is worth highlighting the fact that the estimates of cost of administration and cost of illness are highly uncertain, based on small-scale localised studies. Fortunately, they are not major determinants of the resulting cost-effectiveness estimates.

Finally, while such cost-effectiveness results are an essential input into the decision-making process, they are by no means sufficient. For example, even though this model considers differences in rotavirus mortality in populations of different socio-economic status, it does not weigh the distributional or equity implications of implementing rotavirus vaccination. Because the intervention disproportionally benefits the poorest segments of the population, a government that was seeking to reduce overall inequality in the population might give it higher priority than the simple CE results would suggest. Similarly, political and operational considerations are likely to be very important.

## Conclusion

At prices below US $15 per dose, the cost per life-year saved is estimated to be lower than one GNP per capita and hence highly cost effective according to the suggested criteria of the WHO Commission on Macroeconomics and Health. Cost-effectiveness estimates are highly dependent upon the case fatality in the absence of the vaccine, which suggests that the vaccine is likely to be significantly more cost effective among poorer populations and among those with less access to prompt medical care – suggesting also that poverty reduction programs would be expected to reduce the cost-effectiveness of the vaccine in the future. In other middle-income countries with similar epidemiology (including case-fatality) the results are likely to be similar. However, it is more difficult to extrapolate these conclusions to low income countries. On the one hand, the case fatality is likely to be significantly higher and the cost of administering the vaccine lower because of lower labour costs (although in some countries this will be outweighed by higher transport and logistics costs), both of which would improve the vaccine's cost-effectiveness. Whether this improvement in cost-effectiveness is sufficient to compensate for the much lower ability to pay will depend on the situation in a particular country.

As with any economic evaluation, caution should be exercised in extrapolating the results to other settings. In this case, the results of the modelling exercise are likely to be most sensitive to differences in rotavirus case fatality rates (see Figure 3) because these differ so much among developing countries. Vaccine efficacy is likely to vary relatively little if administered as recommended, but poorer countries are less likely to be able to administer the vaccine at 2, 4 and 6 months, significantly reducing its effectiveness in preventing infant deaths. Other potentially significant factors include differences in cost of adding the vaccine to the current regimen and differences in viral subtypes.

Finally, it is worth highlighting that while rotavirus vaccination represents an opportunity to reduce the burden of diarrhoeal disease in a cost-effective way in the short term, medium to long term solutions such as interventions to enhance health care seeking, education for oral rehydration therapy, health care infrastructure and nutrition improvements, among others, could be highly cost-effective and should be promote by developing countries.

## Competing interests

This study was funded by Merck & Co., Inc., which manufactures the vaccine under the brand name RotaTeq. Robbin Itzler is a full-time employee of Merck & Co., Inc and she also owns stock and holds stock options in the company. Atanacio Valencia-Mendoza, Stefano M. Bertozzi and Juan-Pablo Gutierrez have no other competing interests other than sponsorship of this study by Merck & Co., Inc.

## Authors' contributions

AV–M conceived the study, conducted the analysis of the data, interpreted the results and led the writing of the manuscript. SMB assisted with the study design, interpretation of the results and the writing of the manuscript. J–PG assisted with the study design and with the data analysis. RI assisted with the study design and with the interpretation of the results. AV–M and SMB had full access to all the data in the study and take full responsibility for the integrity of the data and the accuracy of the data analysis. All authors read and approved the final manuscript

## Pre-publication history

The pre-publication history for this paper can be accessed here:



## References

[B1] Rodriguez WJ, Kim HW, Arrobio JO, Brandt CD, Chanock RM, Kapikian AZ, Wyatt RG, Parrott RH (1977). Clinical features of acute gastroenteritis associated with human reovirus-like agent in infants and young children. J Pediatr.

[B2] Sabbaj L, De Petre EE, Gómez JA, Sordo ME (2001). Rotavirus en la diarrea aguda. Arch Argent Pediatr.

[B3] Uhnoo I, Svensson L (1986). Clinical and epidemiological features of acute infantile gastroenteritis associated with human rotavirus subgroups 1 and 2. J Clin Microbiol.

[B4] Parashar UD, Hummelman EG, Bresee JS, Miller MA, Glass RI (2003). Global illness and deaths caused by rotavirus disease in children. Emerg Infect Dis.

[B5] Blanck RE, Greenberg HB, Kapikian AZ, Brown KH, Becker S (1982). Acquisition of serum antibody to Norwalk Virus and rotavirus and relation to diarrhea in a longitudinal study of young children in rural Bangladesh. J Infect Dis.

[B6] Mota-Hernández F, Gutiérrez-Camacho C, Villa-Contreras S, Calva-Mercado J, Arias CF, Padilla-Noriega L, Guiscafré-Gallardo H (2001). [Prognosis of rotavirus diarrhea]. Salud Publica Mex.

[B7] Cook SM, Glass RI, LeBaron CW, Ho MS (1990). Global seasonality of rotavirus infection. Bull World Health Organ.

[B8] Vesikari T, Itzler R, Matson DO, Santosham M, Christie CD, Coia M, Cook JR, Koch G, Heaton P (2007). Efficacy of a pentavalent rotavirus vaccine in reducing rotavirus-associated health care utilization across three regions (11 countries). Int J Infect Dis.

[B9] Marie-Cardine A, Gourlain K, Mouterde O, Castignolles N, Hellot MF, Mallet E, Buffet-Janvresse C (2002). Epidemiology of Acute Viral Gastroenteritis in Children Hospitalized in Rouen, France. Clin Infect Dis.

[B10] Rivest P, Proulx M, Lonergan G, Lebel MH, Bédard L Hospitalisations for gastroenteritis: the role of rotavirus. Vaccine.

[B11] Velázquez FR, Garcia-Lozano H, Rodriguez E, Cervantes Y, Gómez A, Melo M, Anaya L, Ovalle JC, Torres J, Diaz De Jesus B, Alvarez-Lucas C, Breuer T, Muñoz O, Kuri P (2004). Diarrhea morbidity and mortality in Mexican children: impact of rotavirus disease. Pediatr Infect Dis J.

[B12] Velázquez FR, Matson DO, Calva JJ, Guerrero L, Morrow AL, Carter-Campbell S, Glass RI, Estes MK, Pickering LK, Ruiz-Palacios GM Rotavirus infections in infants as protection against subsequent infections. N Engl J Med.

[B13] Secretaría de Salud (2005). Salud México 2004: Información para la rendición de cuentas Mexico.

[B14] Mota-Hernández F, Calva JJ, Gutiérrez-Camacho C, Villa-Contreras S, Arias CF, Padilla-Noriega L, Guiscafré-Gallardo H, de Lourdes Guerrero M, López S, Muñoz O, Contreras JF, Cedillo R, Herrera I, Puerto FI (2003). Rotavirus diarrhea severity is related to the VP4 type in Mexican children. J Clin Microbiol.

[B15] Nafate A (2006). Determinación de costos por caso de diarrea en menores de cinco años atendidos en tres subsistemas de atención a la salud en Morelos, México. MSc thesis.

[B16] República Mexicana: Probabilidades de fallecer por sexo y edad, 2005–2050. http://www.conapo.gob.mx/00cifras/proy/RM.xls.

[B17] Informe sobre desarrollo humano para México 2005 del Programa de las Naciones Unidas para el Desarrollo. http://saul.nueve.com.mx/informes/index.html.

